# A free geometry model-independent neural eye-gaze tracking system

**DOI:** 10.1186/1743-0003-9-82

**Published:** 2012-11-16

**Authors:** Massimo Gneo, Maurizio Schmid, Silvia Conforto, Tommaso D’Alessio

**Affiliations:** 1 Engineering Department, “Roma Tre” University, Via della Vasca Navale, 84, Rome, I-00146, Italy

**Keywords:** Eye-gaze tracking, Human computer interaction, Pupil center corneal reflection, Artificial neural networks

## Abstract

**Background:**

Eye Gaze Tracking Systems (EGTSs) estimate the Point Of Gaze (POG) of a user. In diagnostic applications EGTSs are used to study oculomotor characteristics and abnormalities, whereas in interactive applications EGTSs are proposed as input devices for human computer interfaces (HCI), e.g. to move a cursor on the screen when mouse control is not possible, such as in the case of assistive devices for people suffering from locked-in syndrome. If the user’s head remains still and the cornea rotates around its fixed centre, the pupil follows the eye in the images captured from one or more cameras, whereas the outer corneal reflection generated by an IR light source, i.e. glint, can be assumed as a fixed reference point. According to the so-called pupil centre corneal reflection method (PCCR), the POG can be thus estimated from the pupil-glint vector.

**Methods:**

A new model-independent EGTS based on the PCCR is proposed. The mapping function based on artificial neural networks allows to avoid any specific model assumption and approximation either for the user’s eye physiology or for the system initial setup admitting a free geometry positioning for the user and the system components. The robustness of the proposed EGTS is proven by assessing its accuracy when tested on real data coming from: i) different healthy users; ii) different geometric settings of the camera and the light sources; iii) different protocols based on the observation of points on a calibration grid and halfway points of a test grid.

**Results:**

The achieved accuracy is approximately 0.49°, 0.41°, and 0.62° for respectively the horizontal, vertical and radial error of the POG.

**Conclusions:**

The results prove the validity of the proposed approach as the proposed system performs better than EGTSs designed for HCI which, even if equipped with superior hardware, show accuracy values in the range 0.6°-1°.

## Background

Eye-gaze tracking systems (EGTSs) estimate the Point Of Gaze (POG) of a user. Applications of EGTSs can be classified as *diagnostic*, where the user’s visual and attentional processes are quantified, or *interactive* where the user inter-acts with the EGTS [1]: in the first case, the obtained data are used to study oculomotor characteristics and abnormalities (e.g. in ophthalmology, neurology, psychology); in the second scenario, EGTSs are proposed as input devices for human computer interfaces (HCIs), e.g. to move a cursor on the screen when mouse control is not possible, such as in the case of assistive devices for people with motor disabilities or suffering from locked-in syndrome. Sought-after requirements in EGTSs include minimal intrusiveness and obstruction, reduced calibration phase, allowing free head movements, keeping high the accuracy and the setup flexibility, and maintaining low the cost. Even though there are non-intrusive portable EGTS, many traditional solutions for those systems are intrusive, as they require a physical contact with the user (e.g. contact lenses, reflective dots placed directly onto the eye, electrodes fixed around the eye, bitten and/or head mounted devices). The EGTSs based on *video-oculography* (VOG) (i.e. *video-based* EGTSs), non-intrusively estimate the POG from the information given by the eye images captured from one or more cameras [2,3]. Because of its minimal obtrusiveness, relatively easy set-up and dependence on optical and electronic imaging devices, VOG has become the most popular eye-tracking technique. VOG systems based on visible light are called *passive light*[4], whereas the ones using infrared (IR) are called *active light*. Nowadays, the latters are the most used thanks to numerous advantages: very little subject awareness (users are neither distracted nor disturbed by IR); strong iris reflectance in the near-IR, which grants well-contrasted images irrespectively of iris color, thus easing the pupil detection; low cost, since IR light can be provided by cheap IR light-emitting diodes (ILEDs) and captured by commercial charge-coupled device (CCDs) cameras.

**Figure 1:**
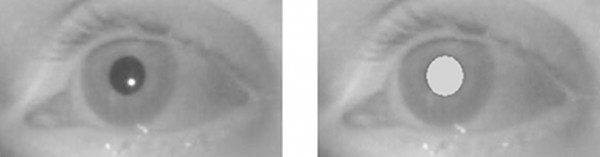
Dark pupil and glint (left), bright eye (right).

The *pupil centre corneal reflection* (PCCR) is the *active light* eye-gaze tracking method par excellence [5]. If the user’s head remains still and the cornea rotates around its fixed centre, the pupil follows the eye in the captured images, whereas the outer corneal reflection generated by an IR light source, i.e. *glint*, can be assumed as a fixed reference point. The POG can be thus estimated from the pupil-glint vector. Both glint and pupil centre locations can be easily extracted from the images captured by a camera under IR light. The glint appears in the IR band as a small intense spot whereas the pupil can be captured thanks to two distinct effects generated by IR: the *dark pupil* (Figure 1, left) if the IR light source is placed away from the camera (*off-axis*), and the *bright eye* (Figure 1 right) if the IR light source is close to the optical axis (*on-axis*) [6-8].

**Figure 2:**
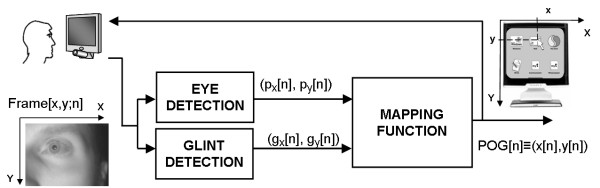
Generic scheme of an EGTS based on pupil centre corneal reflection.

In a generic PCCR-based EGTS (Figure 2) the *mapping function* maps glints and pupil centres in the image onto the POG coordinates. The mapping function is the main typifying characteristic of an EGTS, and is determined through a *calibration* phase during which the user is asked to gaze at a proper set of known points on the observed surface.

The most popular approach for implementing EGTSs is the so called *feature-based* method relating the POG to local eye features such as the pupil and glints for PCCR, the parameters of the system components setup, and the parameters of the eye physiology. Feature-based methods include the *model-based* and the *regression-based* – which we prefer to refer to as the *model-independent* – approaches [2].

The former approach directly estimates the POG by using an explicit implementation of the mapping function derived from geometric models. These models are characterized by physiological and physical parameters related respectively to the user’s eye (e.g. radius of the cornea approximated as a sphere), and to the geometry of the system setup (basically the camera features and the positions of the light sources, monitor, and user’s eye corneal centre).

The *model-independent* approach estimates the mapping function by means of regression techniques, using either parametric (e.g. polynomial) or non-parametric forms (e.g. neural networks), whose coefficients have no physiological or physical meaning.

Both model-based and model-independent methods need a calibration phase to determine the model parameters and the regression coefficients respectively, when the user is asked to gaze at a set of predefined known points on the screen. The analogy between the two approaches is obviously maintained when the conditions move away from the calibration situations and the accuracy quickly decays if the user POG is far from the calibration points.

Model-based methods may simplify – but not avoid at all – the calibration to evaluate the parameters of the model [9,10].

### Model-based methods

We now proceed to argue about the main characteristics and drawbacks of model-based EGTSs.

While the camera and the geometric parameters may be directly measured or estimated once and for all during the first system setup, the physiological parameters of the eye are difficult to measure and are affected by large inter-individualvariability. This makes a calibration phase unavoidable. It was indeed shown that even for an EGTS where the simplified corneal spherical model is adopted and both the camera parameters and the system geometry are perfectly known, the POG determination still needs several physiological parameters, including: the ray of the corneal curvature, the distance between the pupil and the corneal centre, the combined index of refraction of the aqueous humor and cornea, and the angular offset between the optical and visual axes [9,10]. (The *optical axis*, i.e. the eye symmetrical axis, is the line joining the pupil and cornea centre; the *visual axis*, i.e. the gaze direction, is the line joining the POG and the fovea, the highest acuity area of the retina, slightly displaced from the back pole of the eyeball.)

Moreover, it has to be stated that the increase in the number of measurements during the setup (needed for the parameters estimation), decreases the complexity of the calibration procedure as a lower number of calibration points is needed, but at the same time it decreases also the flexibility of the system, as the measured parameters should not be changed.

The values of the parameters provided by the estimation procedure are valid if the EGTS components are left in the same configuration used for the estimation. Any change in the configuration will affect the mapping function and cause an incorrect POG estimation.

The approximation process, that is typical of any model-based method, often makes the model even oversimplified. This is what happens with the ubiquitous corneal spherical model: it is particularly unsuitable for the outer regions of the cornea, where the corneal surface bends towards the sclera [11], leading to high inaccuracy when the user moves the eye to the extremities of the screen and the glint falls onto a non spherical surface. The oversimplification of the model has been reported as one of the main sources of the POG estimation errors [9].

To sum up, the adoption of whatever model-based approach involves the following main drawbacks:

1 the accuracy is limited by the approximation inherent in each model,

2 the initial setup of the system is relatively complex (the model parameters have to be accurately measured), and

3 the system is rigidly bound to the initial setup (once measured, the model parameters must be kept fixed).

Moreover, regardless of the model complexity, the calibration can be only simplified but not avoided at all.

### Neural model-independent methods

The main difficulty with POG estimation is due to the inherent high complexity and nonlinearity of the mapping function, that is particularly severe with large pupil-glint vectors. That difficulty was already faced by model-independent methods by using classical polynomial regression [7] but, as with model-based approaches, the performance quickly decays when POG falls far from the calibration points.

As artificial neural networks (ANNs) – and particularly standard Multilayer Neural Feedforward Networks (MFNNs) – are shown to be universally able to approximate any measurable function to any desired degree of accuracy [12-14], we propose to use MFNNs as a multivariate non-linear mapping to learn the mapping function of a new PCCR-based EGTS.

ANNs are a biologically inspired computational paradigm using many simple elaboration units (*neurons*) highly interconnected. A set of significant inputs and corresponding desired output couples (*training set*) is used to *train* the ANNs connections strengths (*weights*) minimizing the error between the desired and actual outputs [15]. The *generalization* power of ANNs is related with the ability to correctly predict the output value for inputs not contained in the training set. The level of generalization reached at the end of the training is related to both the content of the training set and the complexity of the ANN in terms of the number of neurons and their interconnections. Regarding the training set, the better the (input, output) domains are sampled, the higher is the generalization ability of an ANN; as regards the ANNs complexity, oversimplified ANNs can be unable to identify complicated behaviours (*underfitting*), whereas too complex ANNs may learn the noise affecting the training set data (*overfitting*), becoming unable to correctly behave in conditions far from the contents of the training set.

We speculate in the following about how the appropriate use of MFNNs allows overcoming *both* the drawbacks of the model-based EGTSs *and* the potential reasons of those failures that sometimes gave ANNs an undeserved not-so-good reputation.

The approximation inherent in whatever adopted model may be avoided as ANNs may in principle approximate with the desired degree of accuracy whatever complex EGTS mapping function. Moreover, thanks to their learn-by-examples ability, ANNs may learn any mapping function whatever is the configuration given to the system during the first setup. Therefore, the direct measurement or estimation of the model parameters during the system setup may be also bypassed and implicitly included in the learning of the function mapping, simplifying the setup process itself.

In addition, granted an opportune training set and the right complexity of the ANN, the generalization power of ANNs allows overcoming the problem generally afflicting both model-based and model-independent EGTSs, regarding the accuracy decay when the user’s POG falls on points far from the calibration ones. Uniform accuracy all over the screen may be thus assured.

The above-reported considerations stand if the theoretical behavior of ANNs is hypothesized. Although ANNs have been already used as EGTSs mapping functions [8,16-18] (a brief review will be given in next Section) with large training sets of eye images, the achieved POG estimation accuracy was not as good as for other techniques. Proven that MFNNs are universal and arbitrarily accurate approximating tools, any failure in their application may arise from one or more of the following reasons [13]:

1 lack of deterministic input-output mapping,

2 unmet learning and/or training,

3 improper complexity of the ANN with respect to the problem, and

4 inappropriate choice of inputs.

The adverse situations (2-4) can be avoided if MFNNs are appropriately exploited as mapping functions of a PCCR-based EGTS.

The first topic can be excluded, as the real problem related to the mapping function of a PCCR-based EGTS is not to prove its existence but rather its inherent complexity.

As regards the inadequacy of learning and training, we believe that the EGTS calibration phase is a very good source of data to build an ideal training set. When the user is asked to gaze at a known point, the point coordinates provide the desired outputs, whereas the correspondingly captured eye features provide the related inputs. The training set is built for all the points on the calibration grid, and the codomain of the mapping function corresponds to all the coordinates of the monitor pixels. This output space shows the following interesting properties: it is finite dimensional (2-D), it is bounded with exactly fixed boundaries (the monitor frame), and it has finite cardinality. The codomain of an EGTS mapping function can be thus easily sampled giving a training set that can be arbitrarily made large and uniformly representative of the mapping itself. This is a crucial topic as it is well recognized that overfitting is very dangerous and the best way to overcome it is to build large training sets [19,20].

The last topic, regarding the complexity of MFNNs, implies the selection of the best architecture in terms of number of hidden layers, size of each layer, and interconnections. It is well recognized that this problem is so task-dependent that none of the known methods can be assumed as superior to the others [20]. Though a heuristic trial-and-error approach is often used, especially about the hidden layers, some general rules may be given about the number of input and output neurons. As one of the golden rules of thumb is that the parsimonious architectures have the best performance and the highest generalization capability [19], we believe that the ANNs so far used for POG estimation are too expensive in terms of both complexity and computational cost. An appropriate preliminary phase of eye features extraction on the images should be performed to maximize the compression of the information and minimize its loss, so that the number of ANN inputs is minimized too. This is the approach that has been sought in the EGTS here proposed.

Since ANNs are shown able to learn and approximate mappings from examples to any desired degree of accuracy [21], and we believe that the POG determination is a well posed task for ANNs, we propose to adopt a model-independent approach based on ANNs to overcome the drawbacks of the model-based methods.

While a 1° accuracy is an agreed bound for the specifications of EGTSs designed as input devices for HCIs, we aim at a lower bound of 0.6° in the accuracy, coming from the physiological evidence that in the fovea the highest acuity retinal area ranges from 0.6° to 1° [9].

In [9] a general study for PCCR covering all the possible system configurations in terms of number and positioning of IR sources and cameras is also presented. The multiplicity of glints allows a *theoretical* increase in performance even if this has not been quantified yet.

Provided that with 2 IR sources there is an accuracy of 1° [9], the scope of this work is thus to demonstrate whether the universal regression power of ANNs allows to reach the physiological – and perceivable – lower bound of 0.6° through a 3 IR sources configuration.

The structure of the present work is reported in the following.

Section of Methods describes the theoretical basics of the proposed EGTS, detailing information on each component and showing the setup and the experimental protocol. In particular, the robustness of the proposed EGTS is proven by measuring its accuracy on real data captured from healthy subjects (no subjects with motor disability were used nor any with locked-in syndrome) for different geometric settings of the system setup, considering not only the known points used during the calibration, but *also* halfway test points the user did not cross during the calibration. Details are also provided regarding the performance evaluation metrics used: the proposed EGTS was compared in terms of performance with several model-independent [8,16,17] and model-based [9,10] methods described in literature, which are briefly reviewed together with two commercial EGTSs [22,23].

In the Section of Results, the achieved accuracies are reported and discussed. The proposed EGTS performance met the requirement of 0.6° accuracy and was practically independent on both the system setup and the user. No noticeable training effect in using the system resulted.

As summarized in the concluding Section, the proposed EGTS generally performed better than other above referenced model-independent and model-based methods in literature, approaching the performance of the mentioned commercial EGTSs equipped with superior hardware.

## Methods

### The proposed EGTS basics and components

Reference [9] presented a general study for PCCR covering all the possible system configurations in terms of number and positioning of IR light sources and cameras. Although under general simplifications (corneal spherical approximation, light sources assumed as point sources, cameras assumed as pinhole cameras), some important results were found:

– *1 camera, 1 IR source*: the POG cannot be estimated unless the head is stationary or the head position is estimated by some other means,

– *1 camera, 2 IR sources*: it is the simplest configuration that allows estimating the POG letting the head free.

Under similar assumptions, in [10] it is also shown that:

– regardless of how *many* cameras or IR sources are used, calibration is necessary,

– *1 camera, 2 IR sources*: is sufficient (about 1° of accuracy), whereas the use of more IR sources and calibration points increases the accuracy.

**Figure 3:**
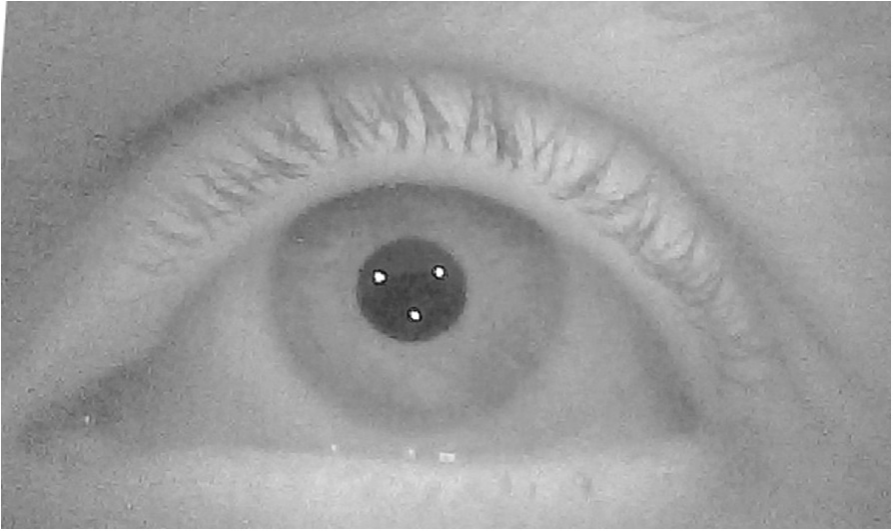
The triangular pattern of the three glints reflected by the eye.

Considered the above results and the need to minimize the number of inputs, we propose to use one camera and to increase the number of IR lights from two to three so that an opportune triangular pattern of glints is projected on the user’s eye (Figure 3), thus allowing the POG estimation even when the head moves. It will be shown in the following that the triangular pattern of glints in Figure 3 allows convenient and robust eye feature detection.

**Figure 4:**
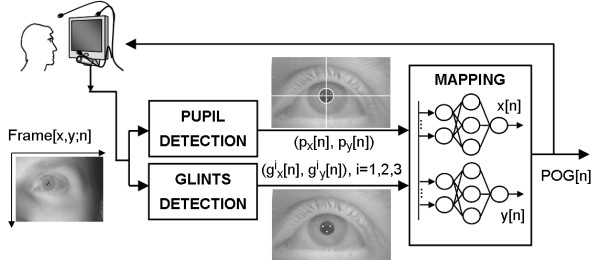
The processing chain of the proposed system.

As depicted in Figure 4, the processing chain of the proposed EGTS starts with two separate blocks extracting the locations of the pupil centre and the three glints, that feed two MFNNs, one for each of the POG coordinates. The MFNNs can be trained for whatever positioning of the user and the system components, allowing to neglect any system or subject-specific eye parameters measure/estimation (free geometry setup). The initial system setup is thus extremely simplified and the following measurements and procedures can be avoided:

– *camera calibration* (the determination of intrinsic camera parameters): any kind of camera can be used,

– *system geometry determination*: IR lights, user, monitor and camera can be freely positioned,

– *monitor measurement*: any kind of monitor can be used, regardless of the resolution and dimension,

– *user’s eye physiology determination*: once the initial setup has been done, the system can be used by different users.

Moreover, whatever change should occur for the system configuration in terms of substitution or positioning of the components, no additional measurements or software modifications are needed. Any constraint to rigidly keep the system invariant after the initial setup may be thus relaxed.

Experimental and simulation results in [9] suggested that even relatively small errors in the estimation of the pupil centre and glints can result in relatively large POG estimation errors. We thus provide in the following some details about the methods used to perform the features extraction.

Two pupil effects are mainly used to detect pupils: the so-called *dark pupil* and the *bright-eye*, which have been briefly described in the Introduction Section. Some solutions use both the mentioned effects requiring two or more on and off-axis light sources be multiplexed in time as well as in the wavelength and/or in the polarization [6-8]. Time multiplexing requires synchronization between the camera frame rate and the light sources activation cycles and causes the POG estimate to be provided at half of the camera frame rate. In addition to the circuital complication due to time multiplexing, other important limitations of the bright-eye effect are: its large variability among subjects; the evidence that from 5 to 10% of people have not sufficiently intense bright-eyes to allow reliable POG estimation [5]; the need to place light sources near the camera axis; and an uncontrolled variability of its effect led by even minor head rotations [2,8].

As other authors did [11], we opted to use only off-axis lighting and dark pupil to estimate the pupil centre, so to avoid the limitations of the bright-eye, reduce the circuital complexity, let free the positioning of the IR light sources and camera, and ease future work including the use of two eyes (the two bright effects will be hardly the same) and let the head free to move.

As the proposed three-ILED approach provides a sufficiently large contrast eye image (the pupil is darker than its surroundings), a simple binary threshold can be successfully applied for the pupil detection. We considered an indoor environment, so that the threshold value has to be initially set for each session and does not need to be adjusted during the same session. After the image thresholding, since we are interested in the centre of the pupil and not in the real pupil shape, the Hough transform for circle center and radius estimation [24] is satisfactorily used as other authors did [11,25]. To decrease the computational burden associated with the Hough transform, the preliminary binary thresholding speeds up the calculation and improves the precision of the pupil centre detection. When no pupil centre comes from the preceding frame, the whole actual frame is processed to find out possible circles. Frames for which the previous pupil centre is available are processed only in a rectangular region of interest, reducing the computational load.

Reference [10] reported that the noise in glint position estimation is due to the glint reduced size, and this brought to the use of two of them. Moreover, the glint detection can be detrimentally affected by artefacts due to the glint rolling off the cornea onto the irregular sclera during large eye rotation [4], daylight, spurious reflections, and non-spherical curvature at the edges of the cornea. We thus propose to use a three-glint pattern, which not only improves the POG accuracy [10], but also adds information by projecting onto the user’s eye a known pattern (Figure 3) that can be used to detect and discard glint artifacts.

The glint detection is solved by a three-stage algorithm: first, the three glints-associated blobs are detected using a binary threshold; second, the centre of mass of each blob is calculated with subpixel accuracy [26], giving the glint candidates; third, some geometric relationships and heuristics related to the triangular reflected pattern are applied to discover and exclude possible artifacts:

– the direction of the three lines joining the three couples of glints candidates must be 0°, 60° and 120° ± some tolerance, otherwise the frame is discarded,

– length of each side of the triangle formed by the glint candidates must fall within a specific range of values, otherwise the frame is discarded.

Despite their simplicity, the verification of the above conditions has been shown very powerful in identifying and discarding spurious glint artifacts.

The subpixel accuracy provided by the detection stages of the coordinates of the three glints and pupil centre is then profitably used in the overall training and neural mapping function.

The pupil centre and glints are indeed used to feed the ANNs in such a way to minimize the number of input neurons. Moreover, in order to minimize the number of output neurons, we propose the use of two separate MFNNs, each one having the same eye features as inputs, with one single output neuron directly estimating one of the X and Y coordinates of the POG. The POG discrete coordinates given by the pixels of the screen will be thus given by the quantization of the two MFNNs output. Regarding the training of the two MFNNs, we propose a rectangular, uniform calibration grid to build an opportune training set, as previously described.

One hidden layer and the standard backpropagation training algorithm are used, whereas the transfer function for the hidden layer and the output units are the hyperbolic tangent (tanh) and the linear function, respectively. The best parsimonious architecture using one hidden layer composed of 10 neurons was heuristically found as the best performing for both the MFNNs. Details on the training phase of the proposed MFNNs are reported in the following Section.

The glints and pupil center mapping onto the POG coordinates is achieved in real-time (future experiments on user trying to follow the contours of an object like a square are planned).

### The experimental setup and protocol

Some EGTSs aim at using low-quality (web) cameras to minimize costs. Low cost solutions with a standard lens may require the camera to be too close to the eye. We thus opted for an analogue B/W video-surveillance camera (FC II Computar, CS mount, Senview varifocal 6-60 mm lens with AutoIris, used near the highest zooming level). The camera is connected to a frame grabber (EASYCAP DC60, 25 fps) through its composite video output. The OpenCV software framework, used to perform the image processing phase, samples each frame giving 640×480 pixels. In front of the camera, a Perspex IR-pass/visible-block filter (wavelengths under 780 nm are blocked) was placed. The overall cost of the described optical system was under 200 € so giving a low cost solution. The triangular off-axis illuminating system was obtained using a three-arm flexible support built with simple twisted wire supporting three groups of four USB-powered ILEDs.

The proposed IR illuminating system provides irradiance well below the recommended 10 mW/cm^2^ safety level [27].

**Figure 5:**
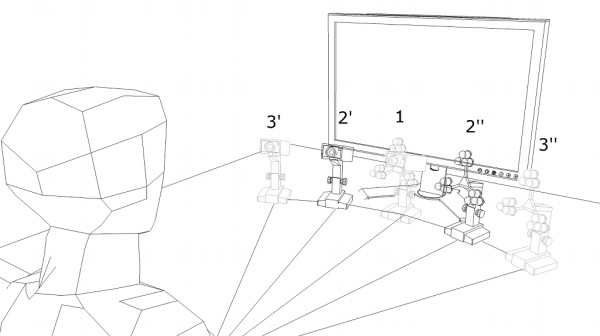
The three different geometric settings for the system setup.

The tests were conducted by positioning the user in front of a 17” monitor (1024×768 spatial resolution and 4:3 aspect ratio). 70 cm far from the user’s eye. In order to assess the independence of the proposed EGTS from the geometry, the accuracy of the POG estimation was evaluated for three different geometric settings depicted in Figure 5. The camera was never calibrated and always placed under the monitor. In the first setting, the triangular lighting system was placed around the camera (see 1 in Figure 5). In the second setting, the camera was placed at an angular distance of 15° to the left of the monitor (see 2’ in Figure 5) and the lighting system was placed 15° to the right (see 2” in Figure 5), so that the overall angular displacement between the camera and IR lights centre was 30°. The third setting was similar to the second one but the overall angular displacement between the camera and IR lights was 60° (see 3’ and 3” in Figure 5).

**Figure 6:**
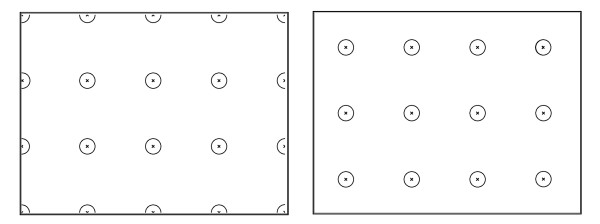
The 4×5 calibration grid (left) and the 3×4 test grid (right).

A 5×4 calibration grid of uniformly spaced points was chosen as it uniformly samples the 4:3 aspect ratio screen (Figure 6, left). The 4×3 test grid is given by the halfway points of the calibration grid (Figure 6, right): it is here outlined that the user’s gaze never crosses the points on the test grid during the calibration. Accuracy was evaluated during both the calibration and the test phases.

Two consecutive test sessions were performed for each of six healthy volunteers, participating to all the three mentioned geometric setting sessions, proposed in random order. Each session directly started asking the user to fix her/his gaze to each calibration point for a fixed period of 1200 ms, corresponding to 30 frames at a capturing rate of 25 fps. During the calibration, the MFNN training set was built collecting only the input given by the estimated centers of the glints and pupil without collecting any image frame. The corresponding desired outputs are given by the coordinates of the known calibration points, whereas ten sigmoidal hidden units are used. The MFNNs training started after the calibration and lasted 1000 epochs. The user was then asked to fix her/his gaze upon each point of a pseudo-random sequence of points on the test grid. Each test point was shown five times, each time for a fixed period of 600 ms (corresponding to 15 frames). The protocol was described to each user, then letting her/him alone and unassisted during the fully automatic overall calibration and test procedure. Each user freely chose the used eye.

Even if the use of ANNs-based mapping functions was shown able to incorporate head movements into the mapping [8,16,17], in this preliminary analysis we opted to defer to future work the tuning and tweaking of the calibration phase to evaluate the performance of the proposed EGTS when users are let free to naturally move their heads. The users were thus asked to keep the head still by means of a head/chin-rest. This also avoided the users to get out from field of view and/or out of focus of the camera.

### Performance measurements and evaluation criteria

**Figure 7:**
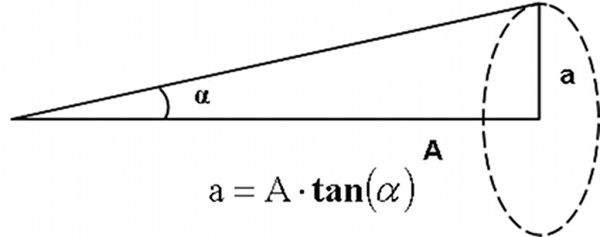
Visual angle trigonometry.

EGTS accuracy, as illustrated in Figure 7, can be expressed in terms of the angular error in visual degrees (smaller angle means higher accuracy) in order to be independent from the screen resolution and distance from the user.

Although the human eye is commonly considered a highly accurate sensor, if used as an input device the exact POG location is inherently not as precise as with a mouse [1].

As a matter of fact, when the user is gazing at a particular point, her/his eyes are oriented in such a way that the POG projects itself on the fovea (the highest acuity region of the retina). Even if during the visual fixation on a still object the POG is perceived as fixed, it is not. This is done to prevent the complete fading of vision, giving blindness during visual fixation [28].

Moreover, the fovea small retinal area is projected onto a finite visual angle (from 0.6° to 1°, [9]) and when we move the eye in order to place the fovea on the area that we want to see with fine details, we do not need to place it exactly centered and on top of the fovea as its projected area becomes larger and hence, covers more the further away an object is [28].

Given the above considerations, it follows that a visual fixation can thus be defined as a stable position of the POG that presents a visual angle dispersion below 1° (foveal area upper limit). POG estimation errors below 1° are thus pursued by most EGTS designers [10].

We may instead add that if the EGTS is designed for HCI it is worthwhile to achieve accuracy under the usually accepted 1° requirement, trying to approach the mentioned lower value of 0.6° for the fovea visual angle. The accuracy shown by the proposed EGTS will be thus analysed in the next Results section considering the lower limit values of 0.6°.

For the generic n^th^ frame during which the user is gazing at one of the known points in of either the calibration or test phases, the quadratic error *e*^*2*^*[n]* between the known point position and the POG estimation was evaluated and accumulated for both the X and Y coordinates. The mean squared error (MSE), and the root mean square error (RMSE) in equations (1) were thus evaluated averaging the error along the N frames of each phase.

(1) $$\begin{array}{l} \text{MS}E_{\text{x}}=\left({\text{Σ}}_{n}e_{x}^{2}\left[n\right]\right)/N,\\ \text{MS}E_{\text{y}}=\left({\text{Σ}}_{n}e_{y}^{2}\left[n\right]\right)/N\\ \text{RMS}E_{\text{y}}={\left(\text{MS}E_{\text{x}}\right)}^{1/2},\text{RMS}E_{\text{y}}={\left(\text{MS}E_{\text{y}}\right)}^{1/2} \end{array}$$

As observed in [10], the results given in terms of the RMSE for X and Y coordinates do not properly measure the POG estimation error. Rather, these errors highlight differences in horizontal and vertical coordinates. The Euclidean distance between the real and estimated POGs in equations (2) should thus be considered as the most representative error value.

(2) $$\begin{array}{l} \text{MS}E_{\text{ρ}}=\left({\text{Σ}}_{n}\left(e_{x}^{2}\left[n\right]+e_{y}^{2}\left[n\right]\right)\right)/N=\text{MS}E_{\text{x}}+\text{MS}E_{\text{y}}\\ \text{RMS}E_{\text{ρ}}={\left(\text{MS}E_{\text{ρ}}\right)}^{1/2} \end{array}$$

The errors in equations (1) were evaluated in terms of pixel difference and then converted into degrees by using the visual angle trigonometry in Figure 7. The Euclidean RMSE_ρ_ was evaluated as in equations (2).

### Other known systems

For the sake of comparison, in this section we briefly consider several relevant EGTSs against which the proposed EGTS was tested. Firstly, we report details about some model-independent EGTSs based on ANNs. Then we describe some model-based methods. Lastly, we shortly review two commercial systems currently giving the *de facto* accuracy lower bounds for EGTSs used as HCI. For simple reference and comparison, the important topics are summarized in Table 1.

**Table 1:** Characteristics and performance of eye-gaze tracking systems and methods

**Cameras**	**Lights**	**Calibration**	**Approach (mapping function)**	**Accuracy**	**Reference**	**Comments/Notes**
1, 20 fps, 640×480	1 (+1)	screen divided in 2×4 zones	MI (2 GRNNs)	5° H 8° V	[8]^abc^	Two concentric IR light rings are alternately turned on and off. Only one glint is used.
1, 30 fps, 640×480	1 (0)	12×16 grid	MI (1 MFNN)	2.4° H 2.4° V	[17]^bc^	Special spectacles frame is needed; both the eyes are used. Accuracy is measured on testing points.
1 low resol.	1	cursor moves	MI (2 MFNNs)	1.5°	[16]^abc^	Accuracy is measured on testing points.
1, 30 fps, 640×480	2	3×3 grid	MB	0.9°	[9]^ac^	
2	2		MB	0.68°	[9]^ac^	Preliminary simulations
1, 60 fps, 640×480	2-4	1 point	MB	≈ 0.7° H ≈ 0.7° V ≈ 1°	[10]^ac^	Each calibration point produces a gaze estimation model. 17 one-point calibrations were performed.
1, 60/120 fps, 640×480	4 (+1)	5 points	NA	0.5°	[22]^ad^	Tracks both eyes simultaneously. Camera and the IR sources are built in the monitor.
1, 60/120 fps	1 (+1)	9 points	NA	0.45°-0.70°	[23]^ad^	Typical and worst accuracy is reported. The POG estimation may require a dedicated computer.
1, 25 fps, 640×480	3	4×5 grid	MI	≤ 0.41° H ≤ 0.49° V ≤ 0.62° E	Proposed EGTS^abc^	Worst accuracy bounds measured on halfway 3×4 testing grid for 3 different geometric settings.

In the reference column, (a) Based on Pupil Corneal Reflection, (b) Mapping function based on artificial neural networks (c) System proposed in scientific literature, (d) Commercial system.

In reference [16], low resolution images were used and all the 600 (40×15) pixels of a rectangular window surrounding the user’s eye are used as input of the two MFNNs. The outputs of each MFNN are respectively provided by 50 units for the X coordinate, and other 50 units for the Y coordinate (the highest output unit represents the estimated coordinate). During the calibration, the user visually tracks a cursor moved in a pre-defined zigzag horizontal path on the screen, and each of the images of the eye is paired with the coordinates of the cursor giving 2000 image/position pairs gathered for training. Other 2000 image/position pairs were also gathered for testing. The best angular accuracy the system achieved on the 2000 testing points was 1.5°.

In reference [17], the user is requested to wear a particular spectacles frame to provide a reference fixed with the head. The EGTS is not PCCR-based and the used lamp is not essential. The coordinates of two points on the spectacle frame, two eyeballs centers and upper and lower eyelids provide the 12 inputs of the used MFNN, whereas the X and Y POG coordinates are its 2 outputs. A 12×16 calibration grid is used and the estimated POG falls almost accurately in a 2×2 square inches window on the screen at distance between 30 and 60 cm (the visual angle trigonometry in Figure 7 gives a best accuracy of about 2.4° in both the directions).

 In reference [8], two identical generalized regression neural networks (GRNNs) – each with a single output unit – estimate the X and Y POG coordinates respectively. The two components of the pupil-glint vector, two coordinates of the single glint, the ratio of the major to minor axes and the orientation of the pupil ellipse provide the 6 inputs of the two GRNNs. During the calibration the user’s gaze was quantized into 8 regions on the screen (2×4 grid) and the same gaze classification was performed by the two GRNNs outputs. The method achieved accuracies around 5° and 8° in the horizontal and vertical direction, respectively.

Some model-dependent EGTSs are now briefly described.

Reference [9] presented a general theory for PCCR-based EGTSs covering whatever cameras and IR light sources number and positioning, under the approximations adopted by most part of the model-dependent EGTSs (IR lights assumed as point sources, video cameras assumed as pinhole cameras, and cornea assumed as a spherical mirror). Test results were reported using a 9 point 3×3 uniform calibration grid for two system configurations, the first using one camera and two lighting sources, the second using an additional camera. Accuracies of 0.9° and 0.68° were respectively achieved.

Under similar assumptions, in [10] a geometric model based on glint positions and pupil ellipse was used to show the minimal required number of cameras, light sources, and user calibration points (user calibration was also shown unavoidable).

We now report some details about the two commercial EGTSs presented in [22] and [23]. Both systems adopt a PCCR method, use ILEDs, remote cameras, implement a software for the overall processing, and require a user calibration to learn the radius of curvature of the cornea and the angular offset between the visual and optical axes of the user.

The EGTS in [22] uses a built-in 640×480 resolution camera capturing two images of the eyes simultaneously at 60 fps or 120 fps producing the respective pupil and glints so providing the EGTS with two different sources of information. Three off-axis light sources are built in the monitor upper frame, whereas a fourth off-axis light source and an extra on-axis light source given by 2 concentric rings of ILEDs are placed around the camera. The EGTS requires a 5 points calibration during which both the bright and dark pupil effects are tested and the best method is chosen. The reported accuracy is 0.5°.

In [23] a 60 or 120 fps camera (no retrievable resolution) is located below the monitor and an ILED at the center of the camera lens generates the glint and the bright pupil. Reported typical and maximum average accuracy is 0.45° and 0.70°, respectively.

The hardware equipment related to both the two mentioned commercial EGTSs appears quite sophisticated and seems to be one of the reasons of their relatively high cost.

For the sake of completeness, the last row of Table 1 anticipates the performance achieved by the proposed EGTS that will be analyzed in the following section.

## Results

The RMSE for the Euclidean, horizontal and vertical coordinates for the three considered system settings are respectively reported in Tables 2, 3, and 4.

**Table 2:** System accuracy - 0° between IR lights and camera

**Session 1**		**Calibration grid**			**Test grid**	
**User**	**RMSE**_**x**_	**RMSE**_**y**_	**RMSE**_**ρ**_	**RMSE**_**x**_	**RMSE**_**y**_	**RMSE**_**ρ**_
1	0.378°	0.303°	0.485°	0.298°	0.526°	0.605°
2	0.336°	0.299°	0.449°	0.308°	0.414°	0.516°
3	0.361°	0.458°	0.583°	0.359°	0.498°	0.614°
4	0.415°	0.434°	0.601°	0.284°	0.612°	0.675°
5	0.414°	0.392°	0.570°	0.330°	0.466°	0.571°
6	0.444°	0.428°	0.617°	0.487°	0.480°	0.684°
mean	0.391°	0.386°	**0.551°**	0.344°	0.499°	**0.611°**
SD	±0.037°	±0.063°	±0.062°	±0.068°	±0.061°	±0.058°
RSD%	±9.4%	±16.3%	**±11.2%**	±19.8%	±12.2%	**±9.5%**
**Session 2**		**Calibration grid**			**Test grid**	
**User**	**RMSE**_**x**_	**RMSE**_**y**_	**RMSE**_**ρ**_	**RMSE**_**x**_	**RMSE**_**y**_	**RMSE**_**ρ**_
1	0.305°	0.512°	0.596°	0.443°	0.516°	0.680°
2	0.387°	0.460°	0.601°	0.421°	0.420°	0.595°
3	0.365°	0.494°	0.615°	0.336°	0.462°	0.571°
4	0.409°	0.351°	0.539°	0.426°	0.436°	0.610°
5	0.425°	0.429°	0.604°	0.482°	0.486°	0.685°
6	0.372°	0.470°	0.600°	0.389°	0.536°	0.662°
mean	0.377°	0.453°	**0.592°**	0.416°	0.476°	**0.634°**
SD	±0.038°	±0.053°	±0.025°	±0.046°	±0.041°	±0.044°
RSD%	±10.1%	±11.6%	**±4.2%**	±10.9%	±8.7%	**±6.9%**
mean	0.384°	0.419°	**0.572°**	0.380°	0.488°	**0.622°**
SD	±0.038°	±0.067°	±0.051°	±0.068°	±0.053°	±0.053°
RSD%	±9.9%	±16.0%	**±9.0%**	±18.0%	±10.9%	**±8.5%**

**Table 3:** System accuracy - 30° between IR lights and camera

**Session 1**		**Calibration grid**			**Test grid**	
**User**	**RMSE**_**x**_	**RMSE**_**y**_	**RMSE**_**ρ**_	**RMSE**_**x**_	**RMSE**_**y**_	**RMSE**_**ρ**_
1	0.308°	0.322°	0.446°	0.333°	0.407°	0.526°
2	0.262°	0.329°	0.421°	0.355°	0.316°	0.475°
3	0.366°	0.424°	0.561°	0.359°	0.480°	0.600°
4	0.317°	0.432°	0.536°	0.355°	0.454°	0.576°
5	0.469°	0.424°	0.632°	0.409°	0.553°	0.687°
6	0.449°	0.395°	0.598°	0.521°	0.468°	0.701°
mean	0.362°	0.388°	**0.532°**	0.389°	0.446°	**0.594°**
SD	±0.075°	±0.046°	±0.076°	±0.064°	±0.073°	±0.081°
RSD%	±20.7%	±11.8%	**±14.4%**	±16.3%	±16.3%	**±13.6%**
**Session 2**		**Calibration grid**			**Test grid**	
**User**	**RMSE**_**x**_	**RMSE**_**y**_	**RMSE**_**ρ**_	**RMSE**_**x**_	**RMSE**_**y**_	**RMSE**_**ρ**_
1	0.249°	0.383°	0.457°	0.381°	0.362°	0.526°
2	0.328°	0.357°	0.485°	0.419°	0.369°	0.558°
3	0.383°	0.450°	0.591°	0.361°	0.436°	0.566°
4	0.306°	0.313°	0.438°	0.526°	0.361°	0.638°
5	0.383°	0.450°	0.591°	0.361°	0.436°	0.566°
6	0.462°	0.371°	0.592°	0.517°	0.419°	0.666°
mean	0.352°	0.387°	**0.526°**	0.427°	0.397°	**0.586°**
SD	±0.067°	±0.049°	±0.067°	±0.069°	±0.034°	±0.049°
RSD%	±19.2%	±12.7%	**±12.8%**	±16.2%	±8.4%	**±8.3%**
mean	0.357°	0.388°	**0.529°**	0.408°	0.422°	**0.590°**
SD	±0.071°	±0.048°	±0.072°	±0.069°	±0.062°	±0.067°
RSD%	±20.0%	±12.3%	**±13.6%**	±17.0%	±14.6%	**±11.3%**

**Table 4:** System accuracy - 60° between IR lights and camera

**Session 1**		**Calibration grid**			**Test grid**	
**User**	**RMSE**_**x**_	**RMSE**_**y**_	**RMSE**_**ρ**_	**RMSE**_**x**_	**RMSE**_**y**_	**RMSE**_**ρ**_
1	0.305°	0.328°	0.448°	0.433°	0.259°	0.505°
2	0.426°	0.382°	0.573°	0.513°	0.495°	0.713°
3	0.279°	0.367°	0.461°	0.347°	0.350°	0.493°
4	0.386°	0.479°	0.615°	0.281°	0.511°	0.583°
5	0.546°	0.352°	0.649°	0.487°	0.429°	0.649°
6	0.537°	0.440°	0.694°	0.488°	0.506°	0.703°
mean	0.413°	0.391°	**0.573°**	0.425°	0.425°	**0.608°**
SD	±0.103°	±0.052°	±0.092°	±0.084°	±0.093°	±0.088°
RSD%	±24.9%	±13.3%	**±16.0%**	±19.8%	±22.0%	**±14.4%**
**Session 2**		**Calibration grid**			**Test grid**	
**User**	**RMSE**_**x**_	**RMSE**_**y**_	**RMSE**_**ρ**_	**RMSE**_**x**_	**RMSE**_**y**_	**RMSE**_**ρ**_
1	0.336°	0.307°	0.455°	0.434°	0.342°	0.552°
2	0.348°	0.291°	0.453°	0.304°	0.388°	0.493°
3	0.298°	0.406°	0.504°	0.289°	0.475°	0.556°
4	0.367°	0.286°	0.465°	0.374°	0.397°	0.546°
5	0.440°	0.358°	0.567°	0.476°	0.474°	0.672°
6	0.433°	0.452°	0.626°	0.524°	0.490°	0.717°
mean	0.370°	0.350°	**0.512°**	0.400°	0.427°	**0.589°**
SD	±0.051°	±0.062°	±0.065°	±0.086°	±0.055°	±0.078°
RSD%	±13.8%	±17.7%	**±12.6%**	±21.5%	±12.9%	**±13.3%**
mean	0.392°	0.371°	**0.542°**	0.413°	0.426°	**0.598°**
SD	±0.084°	±0.061°	±0.085°	±0.086°	±0.077°	±0.084°
RSD%	±21.5%	±16.4%	**±15.7%**	±20.8%	±18.0%	**±14.0%**

The analysis of the results may start from the mean Euclidean RMSE_ρ_: the overall mean RMSE_ρ_ averaged along the users and the sessions for the three system settings (third-to-last rows of Tables 2, 3, and 4) is not only better than the generally accepted accuracy requirement of 1°, but it is also practically always under the limit of the 0.6° lower bound given by the human fovea, as previously discussed. The only exception is the 0.622° RMSE_ρ_ (third-to-last row, last column of Table 2) related to the test grid of the first system setting, that is just slightly above the 0.6° threshold.

That proves the validity of the proposed model-independent approach, in particular if we compare the performance of the proposed EGTS with the accuracy of systems summarized in Table 1. Only the two commercial EGTSs [22] and [23] using superior hardware and rigidly assembled equipment declare a typical accuracy slightly better than the proposed EGTS.

Among the model-based EGTSs, the second system proposed in [9] achieved an accuracy of 0.68°, slightly worse than the proposed EGTS, but two cameras are required. All the other EGTSs reported in Table 1 were less accurate than the proposed EGTS.

The substantial equivalent accuracy shown for all the three system settings also proves the robustness of the proposed EGTS with respect to the geometry of the system setup.

A quite small inter-user Relative Standard Deviation (RSD) of the RMSE_ρ_ is shown for all the three system settings, ranging from the 8.5% of the test grid of the first setting (last row, last column of Table 2), to the 15.7% of the calibration grid of the third setting (last row, fourth column of Table 4). This demonstrates the robustness of the proposed EGTS with respect to different users.

The analysis of the results may follow with the examination of the error statistics related to each session: subjects no. 2 and no. 3 had practiced with the proposed EGTS, while the remaining subjects had no experience with it. Subjects no. 3 and no. 4 were shortsighted, and even if the used eyes required almost 2 diopters of correction, no spectacles were worn during the tests. The performance of each user showed substantial coherence both for the three geometric settings and for the two consecutive sessions (e.g. users no. 1 and no. 2 were generally the best performers, whereas users no. 5 and no. 6 were generally the worst performers).

The overall mean accuracy and the accuracy achieved by single users for the two consecutive sessions are consistent, thus proving the absence of a noticeable learning effect, so that no particular training is required to effectively use the proposed EGTS.

Although the accuracy evaluated on the calibration grid is often slightly better than the accuracy on the test grid, their values may be practically considered as equivalent.

This proves that the proposed EGTS performs uniformly all over the screen and that the training of the ANNs giving the mapping is optimal.

**Figure 8:**
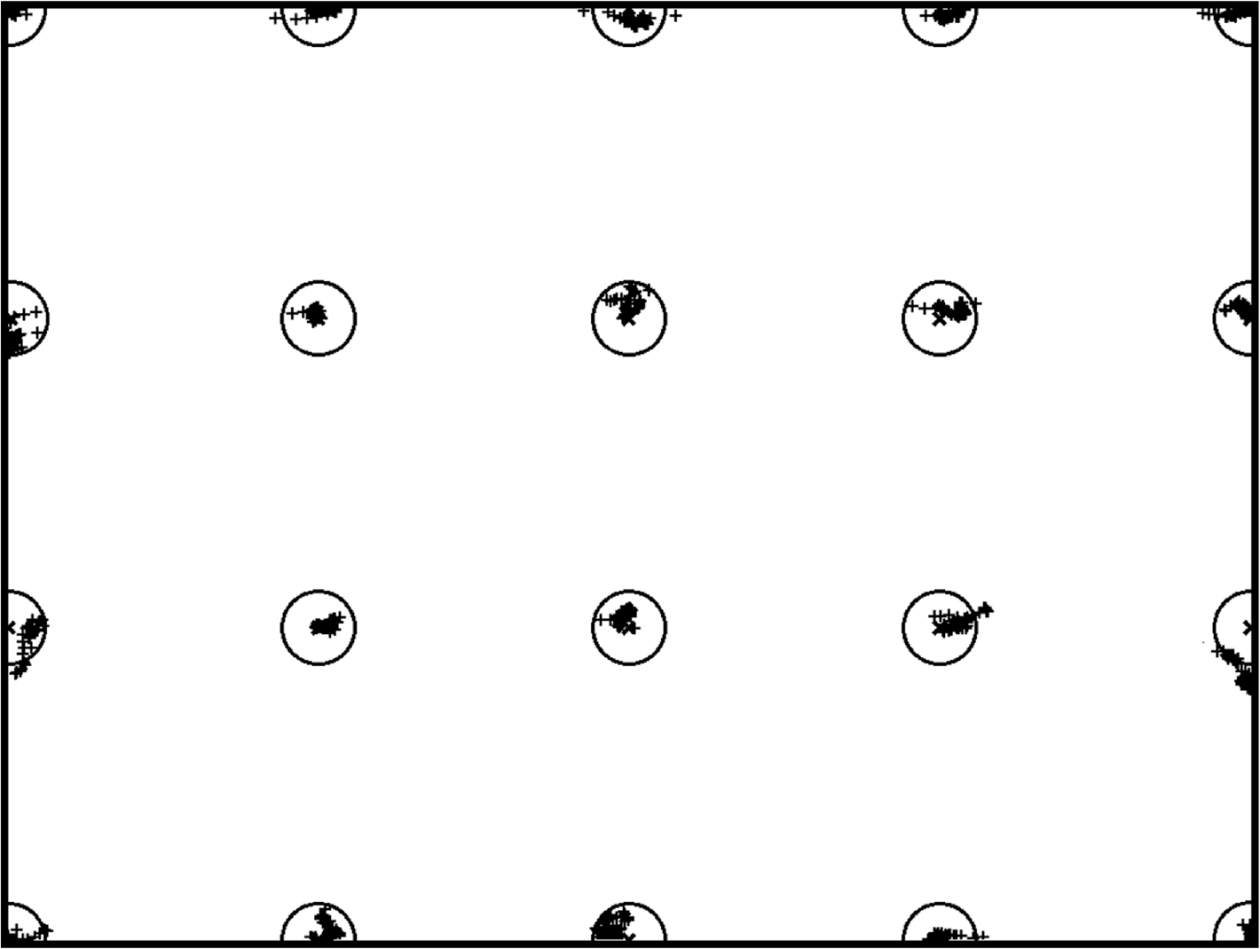
Error distribution on the calibration grid (subject no. 2, 1st session).

**Figure 9:**
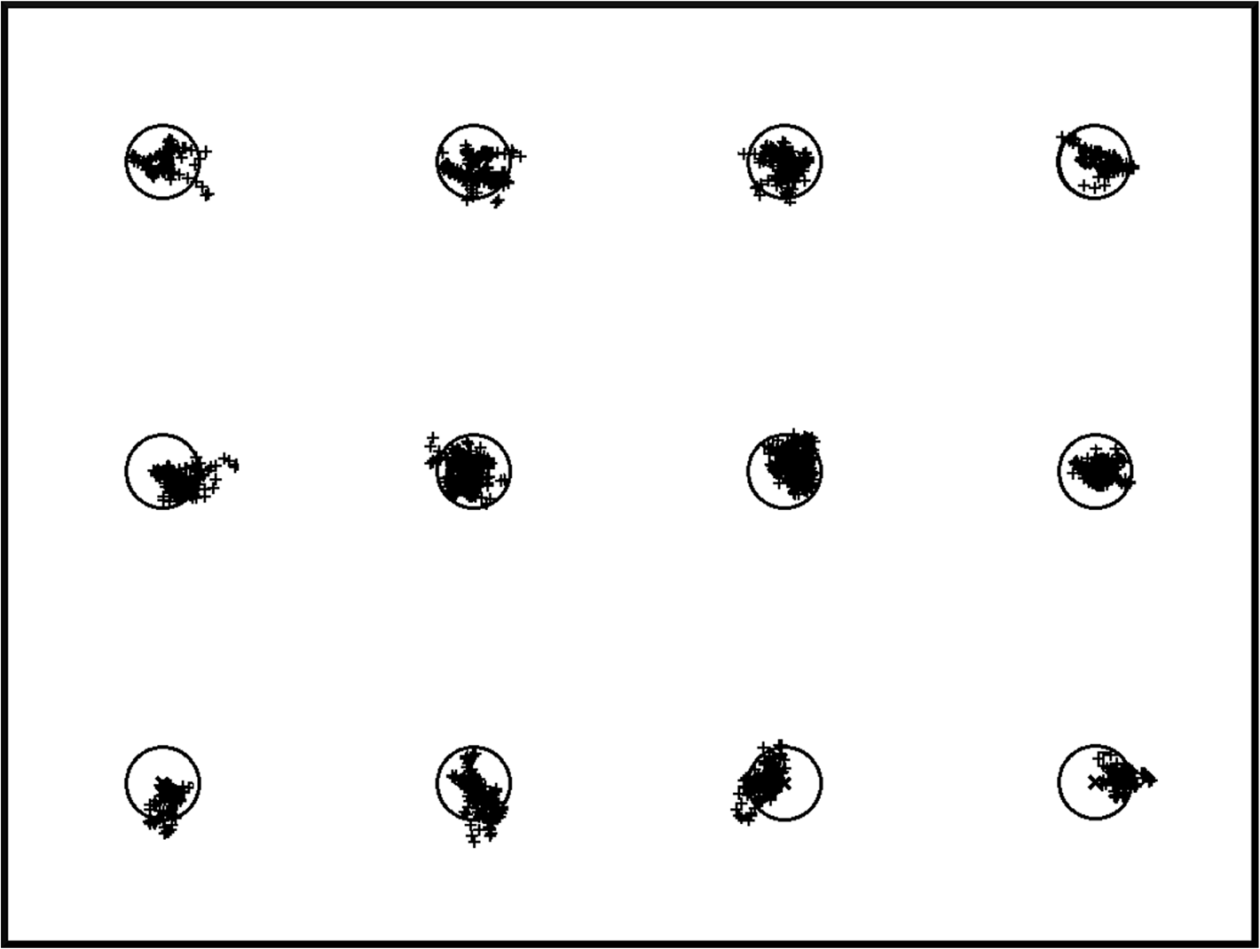
Error distribution on the test grid (subject no. 2, 1st session).

The former point is also shown in Figures 8 and 9, respectively depicting the POG estimation clouds around each correct point on both the calibration and the test grid for the first session of the user no. 2 (please remember that each point on the test grid is randomly shown 5 times, whereas each point on the calibration grid is shown just once). This interesting property grants that the proposed EGTS performs uniformly over the whole screen and does not suffer the quick fall off of the accuracy when the POG moves away from the calibration points as other EGTS generally do.

The point regarding the optimal neural learning grants that the ANNs realized the best approximation of the ideal mapping function. We used one hidden layer MFNN and a trial and error approach to select the parsimonious architecture using 10 hidden units. This architecture showed the results reported in Tables 2, 3, and 4 and required a training time compatible with a real-time operation. Other unreported results regarding using bigger ANNs models (up to 100 hidden units) gave unacceptable training time with no perceivable gain in terms of accuracy.

Good correspondence was shown by the mean RMSE values achieved for the horizontal and vertical errors.

Lastly, performance comparison was also performed against linear regression fitting method for the most challenging configuration (60°). Results, reported in Table 5, showed the superiority of the proposed approach.

**Table 5:** System accuracy - 60° between IR lights and camera Linear regression

**Session 1**		**Calibration grid**			**Test grid**	
**User**	**RMSE**_**x**_	**RMSE**_**y**_	**RMSE**_**ρ**_	**RMSE**_**x**_	**RMSE**_**y**_	**RMSE**_**ρ**_
1	0,351°	0,334°	0,485°	0,433°	0,279°	0,515°
2	0,620°	0,455°	0,769°	0,564°	0,609°	0,830°
3	0,352°	0,416°	0,545°	0,520°	0,325°	0,614°
4	0,472°	0,584°	0,751°	0,576°	0,621°	0,847°
5	0,497°	0,434°	0,659°	0,441°	0,469°	0,644°
6	0,600°	0,529°	0,800°	0,578°	0,590°	0,826°
mean	0,482°	0,458°	**0,668°**	0,519°	0,482°	**0,712°**
SD	±0,106°	±0,080°	±0,118°	±0,061°	±0,137°	±0,128°
RSD%	±22,0%	±17,5%	**±17,6%**	±11,7%	±28,5%	**±18,0%**
**Session 2**		**Calibration grid**			**Test grid**	
**User**	**RMSEx**	**RMSEy**	**RMSEρ**	**RMSEx**	**RMSEy**	**RMSEρ**
1	0,378°	0,373°	0,531°	0,425°	0,460°	0,626°
2	0,502°	0,640°	0,814°	0,472°	0,646°	0,800°
3	0,377°	0,445°	0,583°	0,435°	0,429°	0,611°
4	0,334°	0,307°	0,454°	0,337°	0,371°	0,501°
5	0,458°	0,423°	0,624°	0,482°	0,438°	0,652°
6	0,468°	0,529°	0,707°	0,504°	0,598°	0,783°
mean	0,420°	0,453°	**0,619°**	0,443°	0,490°	**0,662°**
SD	±0,060°	±0,108°	±0,117°	±0,054°	±0,098°	±0,103°
RSD%	±14,3%	±23,7%	**±18,9%**	±12,3%	±20,0%	**±15,5%**
mean	0,451°	0,456°	**0,643°**	0,481°	0,486°	**0,687°**
SD	±0,092°	±0,095°	±0,120°	±0,069°	±0,119°	±0,119°
RSD%	±20,3%	±20,8%	**±18,6%**	±14,4%	±24,5%	**±17,3%**

The glints and pupil center mapping onto the POG coordinates is achieved in real-time and the users did not feel appreciable delay in trying to position their eye gaze onto a target.

## Conclusions

Model-based approach to EGTS is analyzed and its drawbacks highlighted (oversimplified models, complex initial setup, and scarce flexibility of the system after the setup). A model-independent EGTS based on the optimal use of ANNs (training set and complexity of the architecture adequate to the POG estimation task) is proposed and realized. Large flexibility to different users, system setting, and a simplified free geometry setup is allowed, with no need to calibrate the camera and to perform any preliminary estimation or measure. That enables a relatively free engineering of the prototype giving large flexibility to both the assembly of the components and the potential applications.

The proposed EGTS showed also uniform accuracy all over the observed screen and neither particular training nor user assistance was needed.

The worst value of the achieved accuracy (0.622°) is quite better than the requirement of 1° usually accepted to design EGTSs to be used as HCI, approached the lower bound of 0.6° given by the projection of the human fovea, and proved the validity of the proposed model-independent approach.

This lower bound is reached through a combination of effects. First, the multiplicity of glints allows a *theoretical* increase in performance even if this has not been quantified yet. The results obtained thanks to the universal regression power of the proposed ANNs demonstrate that the chosen 3-glint configuration is the simplest configuration that reaches the physiological – and perceivable – lower bound.

Only commercial EGTSs using superior hardware and rigidly assembled equipment declare a typical accuracy slightly better than the proposed EGTS. The latter performs generally better than other examined model-based and model-independent systems.

As the use of ANNs was reported able to incorporate head movement into the EGTS mapping function, we plan to adequate the calibration phase by asking the user to opportunely move her/his head so to measure and achieve good accuracy even when the user is let free to naturally move it.

An open issue about the performance of the proposed system is its ability to work well also when glasses are worn. While a thorough performance evaluation in these cases has not been done in this work, we can anticipate that the flexibility of the system makes it possible to avoid specular reflections on the glasses so that the only limitation would be an attenuation of the corneal reflections.

Future work is also planned:

– to perform experiments on user trying to follow the contours of an object like a square to evaluate the dynamic performance of the proposed system as in [18], and

– to sophisticate the ANNs, for example using feedback connections, which should preserve some of the information from previously estimated eye features and POGs (e.g. recurrent networks), as this would help during blinking [29].

The simplification and optimization of the calibration phase by minimizing the number of points, the gaze duration and the grid structure is another potential field of future investigation.

## Competing interests

The authors have no competing interests to declare.

## Authors’ contributions

MG conceived the proposed approach, developed the related hardware and software, designed the experiments and drafted the manuscript. MS made substantial contribution to supervise and execute the tests and recruitment of the volunteers. MS, SC, and TDA were involved in the interpretation of the results and critical revision of the manuscript. All authors read and approved the final manuscript.
